# New Insights in Staging and Chemotherapy of African Trypanosomiasis and Possible Contribution of Medicinal Plants

**DOI:** 10.1100/2012/343652

**Published:** 2012-04-19

**Authors:** Paul F. Seke Etet, M. Fawzi Mahomoodally

**Affiliations:** ^1^Department of Neurological Sciences (DNNMMS), University of Verona, Via Delle Grazie 8, 37134 Verona, Italy; ^2^Department of Neurology, Yaoundé Central Hospital, Rue Henri Dunant, P.O. Box 87, Yaoundé, Cameroon; ^3^Department of Health Sciences, Faculty of Science, University of Mauritius, Reduit 230, Mauritius

## Abstract

Human African trypanosomiasis (HAT) is a fatal if untreated fly-borne neuroinflammatory disease caused by protozoa of the species *Trypanosoma brucei* (*T.b.*). The increasing trend of HAT cases has been reversed, but according to WHO experts, new epidemics of this disease could appear. In addition, HAT is still a considerable burden for life quality and economy in 36 sub-Saharan Africa countries with 15–20 million persons at risk. Following joined initiatives of WHO and private partners, the fight against HAT was re-engaged, resulting in considerable breakthrough. We present here what is known at this day about HAT etiology and pathogenesis and the new insights in the development of accurate tools and tests for disease staging and severity monitoring in the field. Also, we elaborate herein the promising progresses made in the development of less toxic and more efficient trypanocidal drugs including the potential of medicinal plants and related alternative drug therapies.

## 1. Introduction

Human African trypanosomiasis (HAT) or sleeping sickness is a severe fly-borne disease caused by protozoan of the species *Trypanosoma brucei *(*T.b.*). This disease was first described by European explorers by the late 1800s and early 1900s even if this disease has probably existed in Africa for many centuries [1]. The disease occurs in foci in the tsetse fly (Glossina spp) “belt”, a vast geographical region ranging from the Sahara to the Kalahari Desert equivalent to “the combined size of the United States, India and Western Europe” where these flies have their habitat [2–5]. Three major epidemics of HAT occurred in Africa during the last century, of which the most devastating (which killed millions of persons) occurred from the 1930s to the 1960s [6]. The colonial administrations established mobile teams which systematically screened people in the endemic areas, curing those found with the disease. This initiative resulted in a significant roll back of the disease. In the early 1960s, HAT ceased to be a public health problem, and was no more considered [7]. From the 1970s to the 1990s, favored by dramatic events such as wars and population movements, HAT re-emerged and became an ongoing epidemic. WHO, private partners, and local governments took action, resulting in a significant decrease of the number of new cases reported which, in 2009, which was lower than 10,000 for the first time in 50 years [6].

**Figure 1:**
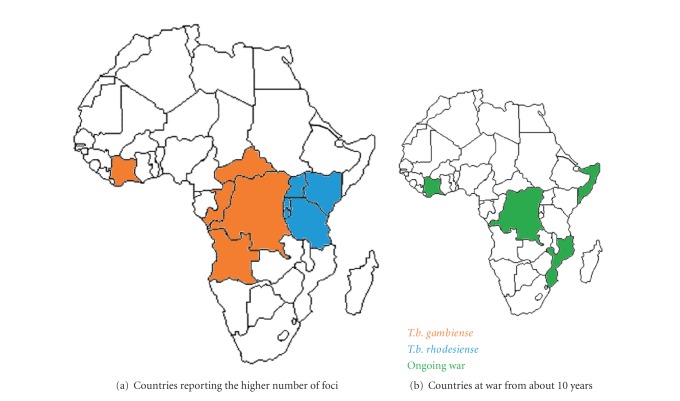
African trypanosomiasis repartition and sociopolitical instability. (a) Illustration of the geographical repartition of the countries reporting the higher number of foci of both *T.b.* subspecies causing HAT. (b) Illustration of the geographical repartition of the countries at war from more than 10 years. Note the correlation between countries at war and the localization of foci of *T.b. gambiense*. HAT cases occur more often in countries with conflict, high political terror, or civil war, with a lag of about 10 years between the conflict beginning and peak in incidence [12]. Epidemiological data are from [16].

Despite these encouraging results, HAT is still a considerable burden for life quality and economy in many sub-Saharan Africa countries, where there may be 200 foci and 15–20 million persons at risk [8], as a large number of new infections may remain unreported or undiagnosed because of remote accessibility of many areas of the endemic region and ongoing wars [9–11]. Besides, it is generally assumed that new epidemics of HAT could occur, originating from these uncontrolled areas where there still are very active foci [12], as illustrated in Figure 1. HAT affects poor and remote rural populations dependent on agriculture, fishing, or hunting. Until very recently, this disease was receiving very few attention, and health interventions and research and development were inadequate to the need [6]. In the last 50 years, only one drug, eflornithine, has been developed even though a huge amount of knowledge of African trypanosome biology has been accumulated in the meantime [13]. Overall, the current drugs used to cure HAT are expensive, highly toxic, need parenteral administration, and parasites increasing resistance has been observed [14, 15]. Therefore, less toxic, more efficient, easy-to-administer and nonexpensive drugs are urgently needed in the field. WHO and some private partners have been recently multiplying initiatives, offering funding for research activities for this purpose. Some encouraging results have already been reported. The research activities have been also aiming at developing new field suitable, easy to-use, and cheap tools to solve the HAT diagnosis, staging, and follow-up issues observed in the field.

## 2. Diagnosis

HAT occurs in two forms: the Gambian or West African form (caused by *T.b. gambiense*) and the Rhodesian or East African form (caused by *T.b. rhodesiense*). The two forms differ in their clinical course, which is chronic (with a course from months to years) in the Gambian form, which represents about 95% of cases, and acute or subacute (with a course from weeks to months) in the Rhodesian form, which represents a minority of cases [7, 8]. After infection and a relatively long (Gambian form) or short (Rhodesian form) latency time, HAT evolves in two stages: a hemolymphatic stage (first stage or stage 1), which develops into a meningoencephalitic stage (second stage or stage 2) irreversibly followed by death if untreated [17, 18]. The hemolymphatic stage entails bouts of fever, headaches, adenopathy, joint pains, and itching. The trypanosomes proliferate at the site of infection and then spread to the draining lymphatic network and bloodstream of the host, where they continuously multiply and from which they invade the peripheral organs. Evidence from animal models shows that at this stage of the disease, the parasites already reside in the brain, but only in the structures located outside the blood-brain barrier (BBB) such as circumventricular organs [19]. The meningoencephalitic stage starts when the trypanosomes cross the BBB and invade the central nervous system parenchyma, excluding then the possibility to cure patients with drugs used in the first stage, as they do not cross the BBB in amounts sufficient to kill the parasites. The second stage is marked by a complex neuropsychiatric syndrome characterized by changes of behavior, confusion, sensory disturbances, poor coordination, a disruption of the sleep-wake cycle, and an alteration of the sleep structure [20, 21]. These qualitative alterations of sleep gave to HAT its alternative name of sleeping sickness. While the drugs in use to cure the first stage are relatively safe, the drugs in use to cure the second stage are highly toxic, and resulting undesired effect include death in about 5% of cases [21, 22].

In a general way, the clinical features of HAT do not suffice for a precise diagnosis, and HAT is usually confused with another endemic and more frequent sub-Saharan Africa disease: malaria caused by the apicomplexan parasite *Plasmodium falciparum* [23]. Therefore, For HAT diagnosis in the field, physical examination for posterior cervical lymphadenopathy (Winterbottom's sign) is performed, and a venous blood sample is taken from the subjects [24]. The blood is screened for the presence of specific antibodies against the parasite with the card agglutination test for trypanosomiasis (CATT). CATT is used in the field for mass screening of HAT because of its sensitivity and ease of use [24, 25]. However, CATT is not specific. False-positive results have been reported in areas of low endemicity [26], and in several foci in West Africa were found some *T.b. gambiense* strains lacking the gene that encodes the surface glycoprotein LiTat 1.3 [27], main gene targeted by the agglutination process [28, 29]. To solve this problem, in addition to CATT are performed microscopic examinations of lymph aspirated from enlarged cervical lymph nodes or of blood films to assess the presence of the parasite in the lymph or blood [30]. If the CATT remains positive at a dilution of 1 in 8 or greater and trypanosomes are seen in the blood film (or lymph), the subject is diagnosed with HAT [31, 32]. However, the occurrence of cases with positive CATT without parasites is common, and considering the high toxicity of the trypanocidal drugs (even those used to cure the first stage), the management of such cases is debated and constitutes an important issue [33]. Therefore, as possible replacement of low sensitivity current parasite detection methods has been suggested, molecular methods are far more sensitive [33, 34].

Generally, *T.b. gambiense* loads in the blood are low, and molecular methods request concentration techniques to increase the detection of these parasites [25]. Such techniques include capillary tube centrifugation, quantitative buffy coat, and minianion exchange centrifugation technique (mAECT) [35, 36]. The latter was recently improved and is the most sensitive technique for trypanosome detection of the blood [37]. The mAECT technique consists in the separation of trypanosomes by anion exchange chromatography on diethylaminoethyl cellulose and low-speed centrifugation to concentrate the eluted trypanosomes. The parasites can then be detected by direct microscopic examination of the sediment in a transparent collector tube [25, 33]. This test presents the advantage of applicability in the field conditions and is robust and less cumbersome than previous versions which required mounting a collector tube in water for microscopic examination [38]. However, the main bottleneck and issues presented by this test as presently formulated are the need of qualified personnel to perform it [39, 40], the short-time stability (1 year maximum at 37°C), as glucose is incorporated in the column buffer [25], and the need of very specific apparatus rarely found in the hospital of rural areas where the disease is endemic [41].

Another attempt to characterize the HAT status of CATT-positive subjects is represented by the “immune trypanolysis test,” a technique assessing the absence of nonspecific trypanolytic activity in the plasma [26]. Studies evaluating this technique were performed on plasma collected from CATT-positive subjects with diverse epidemiological status identified during medical surveys in Guinea, Ivory Coast, and Burkina Faso HAT foci. This test appeared to be a marker for contact with *T.b. gambiense*, suggesting its possible use as a tool in the field to identify nonparasitologically confirmed CATT-positive subjects as well as those who had contact with *T.b. gambiense* and should be followed up [26].

Also of interest are the polymerase chain reaction (PCR) and nucleic acid sequence-based amplification techniques modified by coupling to oligochromatography for easy and fast visualization of products [33]. These techniques appeared to be very sensitive and specific for diagnosis of *T.b. gambiense* in studies performed on blood samples from DRC HAT patients. However, they failed to be as sensitive and specific for *T.b. rhodesiense* detection on blood samples from Uganda HAT patients [42].

Of high interest for the development of less invasive HAT diagnosis tests are the encouraging results from studies performed on saliva samples from *T.b. gambiense* HAT patients using optimized test formats on the basis of enzyme-linked immunosorbent assay (ELISA) antibody detection technique. As ELISAs performed on serum and CATT performed on whole blood or serum, ELISAs performed on saliva appeared to be more than 90% sensitive and specific for the detection of trypanosome-specific antibodies in the saliva [43–45]. Contrarily to CATT which cannot be successfully performed with saliva due to its insufficient analytical sensitivity and the occurrence of unspecific agglutination reactions, ELISA presents the advantage of a high specificity [38]. Unfortunately, ELISA is not applicable for mass screening of the population at risk in sub-Saharan Africa rural areas, as this technique requires large volumes of pure water, pipettes, and many secondary antibodies and conjugates that are not stable at ambient temperatures [46]. Besides, the test takes a few hours [45].

Overall, there is still no accurate serological screening test for *T.b. rhodesiense *infection in the field, where the same tests used for *T.b. gambiense *are used with less accurate results, and unfortunately, there is no promising laboratory breakthrough to solve this problem. On the other hand, powerful molecular techniques have been successfully tested for the diagnosis of *T.b. gambiense* HAT. However, up to now, many of these techniques still need to be modified and adapted to field conditions in order to reach the patients. The development of simple and standardized tests applicable to field conditions from these findings is considered [33].

## 3. Staging and Posttreatment Followup

As previously stated and discussed in Section 2, the drugs used to cure the HAT first stage poorly cross the BBB, and other drugs, which are far more toxic, are used to cure stage 2 patients. Therefore, after HAT diagnosis, the disease stage determination is a crucial step to decide the treatment to administer. After treatment, patients must be followed up to early detect and cure the relapses.

### 3.1. Who Criteria

The commonly accepted criteria in used in the field for HAT staging are from 1998 WHO recommendations [47] modified in 2006 [31]. According to these recommendations, HAT stage 2 is marked in the CSF by the presence of trypanosomes, by alterations of the total protein level (different cutoffs have been proposed and vary from 250 to 450 mg/L), and by elevated white blood cell (WBC) counts (cut-offs are Stage I < 5 cells/*μ*L, “intermediate” stage with 5–20 cells/*μ*L, early Stage II at 20 cells/*μ*L) [22, 38]. The study of cells in the CSF of HAT patients for disease staging was justified by the postmortem findings [10]. However, WHO cut-off criteria seem to have been decided arbitrarily, resulting in malfunction of these criteria in the field [48]. Overall, these criteria are debated [7, 11, 17], and African trypanosomes are rarely found in the CSF of patients, even in the late HAT stage 2 [32, 48].

For posttreatment followup, WHO recommends that treated HAT patients be followed for up to 2 years before a decision on treatment outcome can be taken [49]. Treated patients' blood and CSF are to be examined every 6 months [22, 43]. However, due to the generally low sensitivity of the available parasite detection tests, a substantial number of relapsing patients is not detected early and thus not given treatment. This results in prolonged suffering and even death of patients and also has major consequences at the community level, because infected people act as reservoirs for *T.b. gambiense* [25]. Additionally, many patients are afraid of lumbar puncture and as soon as they feel better, they cease coming to follow-up appointments [17, 23].

Therefore, less invasiveness and better criteria for disease staging are needed, considering the importance of such criteria for the treatment and followup of patients. Less invasive and sensitive diagnostic tools are also needed for disease severity monitoring for relapses detection and management.

### 3.2. Proposed Markers and Tools for HAT Staging and Followup

#### 3.2.1. Infiltrating Inflammatory Cells

Knowledge about the inflammatory cells infiltration and brain damage in HAT originates mostly from animal studies, as only a few clinical studies reporting observations on deceased HAT patients have been published [10]. In *T.b. gambiense*-infected vervet monkeys, perivascular cuffing, meningitis, and encephalitis have been described, with inflammatory infiltrate comprised mononuclear cells, lymphocytes, plasma cells, and Mott cells [50]. In this model, as well as in *T.b. rhodesiense*-infected mice and in *T.b. gambiense*-infected rats, trypanosomes appeared to spread together with inflammatory cells, being first located in the choroid plexus, then spreading to the perivascular space and finally to the brain parenchyma, resulting in a triphasic meningoencephalitic inflammatory disease [51, 52]. The 3 phases which have been envisaged are (i) a chronic meningitis with plasma cells, lymphocytes, and monocytes in the subarachnoid and pial connective tissue, (ii) a progressive neuroinflammation from the meninges to the cerebral vessels entering the brain, and (iii) the development of encephalitis.

Overall, the human post-mortem material examination has revealed a pattern of neuroinflammation similar to that observed in animal models though with some differences in the severity of inflammatory reaction features [21]. The hallmark of CNS pathology in autopsies of HAT fatal cases is a generalized meningoencephalitis with marked cellular proliferation seen in the leptomeninges together with diffuse perivascular infiltration of white matter with lymphocytes, plasma cells and macrophages, and activated macrophages and astrocytes within the perivascular cuffs and adjacent parenchyma [53].

To enter the brain parenchyma, *T.b.* as well as WBC cross the BBB. This physical barrier situated between the lumen of the cerebral blood vessels and the brain parenchyma is formed by tight junctions of the endothelial cells of blood vessel walls surrounded by basement membrane and astrocyte endfeet [54, 55]. To cross the BBB and enter the brain parenchyma, leukocytes establish loose connections with endothelial cells via selectin-integrin interactions, which allow them to roll along the endothelial cell barrier with the flowing blood. Leukocyte transmigration occurs in response to the presence of surface-bound luminal chemokines following a chemotactic gradient. If these chemokines are fixed by leukocyte chemokine receptors, signaling pathways within the leukocyte are activated resulting in conformational changes in the leukocyte integrins, leading to high-affinity binding to the endothelial cell via adhesion molecules. Then the leukocytes move to the inter-endothelial junction, and through that junction, they extend protrusions, sampling for abluminal chemokines. After crossing the endothelial cell layer, leukocytes are sequestered in the perivascular space between the endothelial cell basement membrane and the parenchymal basement membrane. For the completion of the transmigration into the brain parenchyma, the degradation of the cellular matrix by matrix metalloproteinases is needed [56, 57].

In the presence of elevated amounts of the proinflammatory cytokine tumor necrosis factor-(TNF-)alpha, the binding of leukocytes to cellular adhesion molecules and their transmigration across the blood-CSF barrier are increased [58]. This cytokine level is high all over the course of African trypanosomiasis, and this finding could explain at least part of the observations made on the brains of deceased HAT patients. On this basis, studies were recently performed in the blood and CSF of *T.b. gambiense* HAT patients in Angola and Gabon to determine the number and types of leukocyte immunophenotypes present along the disease course. From this studies emerged that the number of B cells in the CSF could be a good indicator of HAT stage and disease severity [59]. Other studies, on basis of the investigation of the CSF from *T.b. gambiense* patients at different stages of infection, have also suggested the amount of B cell in the CSF as indicator of HAT stage and severity [60]. Even if the application of this approach would still require the invasive and “frightening” lumbar puncture, B cells rosettes are easily detected in field conditions [60] and would therefore constitute a good replacement for WBC count.

#### 3.2.2. Inflammatory Mediators

The precise mechanisms by which *T.b.* enter the brain and how this parasite and the infiltrating inflammatory cells interact between them and with resident cells to produce the alterations resulting in the specific meningoencephalitis observed in African trypanosomiasis are still to be unraveled. However, the proinflammatory cytokine interferon- (IFN-) gamma is likely to play a critical role for the traversal of the BBB by *T.b.* [61, 62].

Numerous studies in animal models indicate that the expression of inflammatory mediators (cytokines, chemokines, and adhesion molecules) change with the course of the infection, with a central role played by the balance between pro- and anti-inflammatory mediators in the outcome of the disease (see Kristensson et al., 2010 for review). Findings have pointed to an association between cytokine expression, particularly IFN-gamma and TNF-alpha, and the onset and development of the neuroinflammatory reaction. The CSF levels of the chemokines CXCL-2, CCL-5, CCL-3, and CCL-2 have been reported to increase in the brain early after infection; the early source of these inflammatory mediators appeared to be the brain resident cells astrocytes and microglia, with T cells and macrophages taking the production over later during the disease course [10, 63]. This finding suggests that the initial steps in the development of the neuroinflammatory disease are controlled from within the CNS. Such factors may be responsible for initiating inflammatory cell and *T.b.* infiltration to the brain parenchyma, that is, the beginning of African trypanosomiasis stage 2 [51].

In contrast to the cytokine profiles derived from rodent models, no significant changes in TNF-alpha or IFN-gamma CSF concentrations were reported in humans [43, 64]. Such a discrepancy could reflect either divergences between the cytokines present in the brain and the CSF, or variations in the sensitivity of the assay systems used [51].

In terms of clinical data, a correlation between IFN-gamma concentration in the plasma and disease progression in the CNS has been shown in HAT patients in Uganda (*T.b. rhodesiense*), but no significant changes were found in CSF levels of TNF-alpha or IFN-gamma [64, 65]. In the CSF of these patients were also found significant increases of interleukin- (IL) 10 and IL-6 levels. In DR Congo *T.b. gambiense* HAT stage 2 patients, increases of IL-6, IL-8, and IL-10 in the CSF have been reported; the levels of these cytokines were found to be reduced after drug treatment and investigation of serum/CSF concentration quotients indicated an intrathecal synthesis of IL-10 in 29% of patients [65, 66].

On the basis of the hypothesis that brain damage and inflammation-related proteins could individually or in combination indicate the CNS invasion by* T.b.*, many studies aiming at the determination of markers for efficient discrimination of the HAT stages have been recently searched in the CSF by proteomic analyses. CSF samples from *T.b. gambiense* patients, diagnosed on the basis of CSF WBC counts and presence of parasites, have been used to study the levels of 3 brain damage-related proteins (H-FABP, GSTP-1, overexpressed in post-mortem CSF, and S100b, marker of BBB and neuronal damage) and 13 inflammation-related proteins (IL-1-alpha, IL-1-beta, IL-6, IL-9, IL-10, G-CSF, VEGF, IFN-gamma, TNF-alpha, CCL2, CCL4, CXCL8, and CXCL10). The findings indicated that CXCL10 could distinguish stage 1 from stage 2 patients, with a sensitivity of 84% and 100% specificity, and a panel characterized by CXCL10, CXCL8, and H-FABP was defined to improve the detection of HAT stage 2 patients [48]. These analyses were performed on a relatively limited sample of patients from the same cohort, and still are to be validated in a larger multicentric cohort, but other experimental evidence from animal models and HAT patients confirmed these findings [67, 68].

## 4. Treatment and Vaccine Development

### 4.1. Presently Available Drugs

Four trypanocidal drugs are mainly in use in the field: pentamidine and suramin, which are efficient in the early stage of the disease, and melarsoprol and eflornithine, which are efficient in the late stage. The field drugs, particularly those used in the second stage of the disease, have severe side effects and may even be fatal [14, 32, 69, 70].

#### 4.1.1. Suramin

Pioneering work of the German researcher Paul Ehrlich (1854–1915), winner of the Nobel Prize in Physiology or Medicine in 1908, demonstrated that naphthalene dyes, trypan red, and trypan blue have trypanocidal activity due to selective accumulation by trypanosomes. Following Ehrlich's observations, suramin, a colorless polysulphonated symmetrical naphthalene derivative drug, was developed in the 1920s [71]. This drug has also been used against the filarial parasite *Onchocerca volvulus*, and trials against human immunodeficiency virus, and other human viruses, and against different types of cancer have been performed [72, 73]. A typical protocol of 5 slow intravenous injections, every 3–7 days, over a 4-week period, is used to cure HAT [14, 69].

The trypanocidal action of suramin is still unclear, and many hypotheses have been proposed. (i) Suramin could impede uptake of serum proteins or inhibit endocytosis and key enzymes in metabolic pathways such as glycolysis thanks to its negative charge and the chemical properties derived [74]. Thus, suramin could act by the formation of complexes with LDL impeding the receptor-mediated uptake of LDL, carrier of cholesterol required for parasite growth. (ii) Suramin could accumulate inside the lysosomes and inhibit some key enzymes such as 3′-nucleotidase or protein kinase (which both bind to the plasma membrane of the trypanosome), acid phosphatase or acid pyrophosphatase (in the flagellar pocket), or phospholipase A_1_. (iii) Suramin could also inhibit the high positive-charged glycolytic enzymes located inside the glycosome on the African trypanosomes [69, 75].

As African trypanosomes are unable to synthesize *de novo* fatty acid and cholesterol, the development of resistance to suramin in the field is unlikely considering the important role of LDL in the growth and proliferation of these parasites [69, 75]. However, reports of treatment failures from foci of the Gambian form of HAT in the 1950s led the use of this drug mainly for the Rhodesian form of HAT [14]. In veterinary use, resistance has been noted in some trypanosome species, such as *T. evansi *[76]. The mechanisms of resistance are still to be unraveled.

A considerable amount of suramin binds to serum proteins, and consequently, the suramin half-life in serum is very long (44–54 days in the study of Collins et al., 1986). Although HAT regimens are considered short enough to offer safety and tolerability, the US Food and Drug Administration blocked the approval of suramin for use in prostate cancer because of the adverse effects reported [69].

#### 4.1.2. Pentamidine

Pentamidine (1,5-bis (4-amidi-phenoxypentane]) is a diamidine, that is, an aromatic diamine, which has been used for several decades in the chemotherapy of African trypanosomiasis, leishmaniasis, and against *Pneumocystis carinii* pneumonia in acquired immunodeficiency syndrome patients [69].

As for suramin, the pentamidine mode of trypanocidal action remains uncertain. Overall, diamidines act directly against the parasites independently of their physiological action against the host, and the transport of these drugs across the cell membrane is a necessary first step to antiparasitic action [69, 70]. The trypanosomes accumulate large amounts of pentamidine via P2 aminopurine permease [77]. In trypanosomatids of the *Leishmania* species, close relatives of trypanosomes, fluorescent analogues of pentamidine have been shown to accumulate mostly in the mitochondria resulting in the permanent damage of these organelles and cell death [78, 79]. In addition, in Leishmania, the pentamidine resistance correlates with a reduction in the mitochondrial membrane potential [80, 81]. This is due to the fact that pentamidine interacts electrostatically with cellular polyanions, binding DNA including the kinetoplast. This latter organelle is a characteristic of kinetoplastid flagellates and is constituted by a unique intercatenated network of circular DNA molecules which make up the mitochondrial genome [82, 83]. However, whether the localization of fluorescent analogues of pentamidine correlates with activity is not certain, and, in addition, the mammal bloodstream form of *T.b.* can survive kinetoplast DNA disintegration [84].

A high-affinity and a low-affinity pentamidine transporter (HAPT1 and LAPT1, resp.) contribute to pentamidine uptake. These transporters explain, at least in part, the efficacy of this drug also against melaminophenyl arsenical-resistant parasites that lack the P2 transporter [70, 79]. A retained activity of the P2 transporter has been shown in an African trypanosome laboratory line selected for pentamidine resistance [69, 77]. Furthermore, lack of HAPT1 transporter has been observed in another pentamidine resistant line also lacking the P2 transporter [85]. On this basis, it has been suggested that the resistance to pentamidine may be due to the lack of pentamidine transporters [14]. However, as these pentamidine-resistant lines displayed much reduced virulence in rodent models, it has also been suggested that the development of resistance to pentamidine is associated with substantial fitness costs, therefore rendering the propagation of resistant lines in the field unlikely [14, 70].

#### 4.1.3. Melarsoprol

The mechanism of action of the arsenical compound melarsoprol has been recently reviewed [13, 86]. This drug is still the most widely used to cure the late stage of HAT despite its extremely toxic side effects [14], as it is the only drug effective in the second stage of the Rhodesian form of HAT, and as it is far less expensive than the other drugs used in the second stage of Gambian form of the disease [22, 31, 76].

The uptake of melarsoprol in the trypanosomes is accomplished by purine transporters, as this drug acts as a competing ligand for the purine site on the transport protein [14]. Purine transport is highly developed in trypanosomes as they directly acquire nucleic acids from their hosts. The trypanosomes lyse rapidly when exposed to melarsoprol [87]. *T.b.* thiol-containing enzymes (such as glycerol-3-phosphate dehydrogenase) could be the targets of melarsoprol, as it has been reported that trypanocidal analogues of this drug (such as cymelarsan used to treat nagana) bind strongly to these enzymes [88]. Functional alterations of these enzymes could underlie the lysis of trypanosomes, as they lead to inhibition of glycolysis, and therefore to the loss of ATP, although these cells seems to lyse before ATP supplies are seriously depleted [14, 87].

The active metabolites of melarsoprol contain a trivalent arsenic element with a markedly reactive arsenoxide group, which confers the physicochemical ability of lipid solubility that allows the passage of the drug across the BBB [89, 90]. In addition to this transport function, the arsenoxide group probably mediates the killing of trypanosomes in the cerebrospinal fluid (CSF). This is suggested by the fact that modifications of the melarsoprol parent ring have a significant impact on its trypanocidal action. The trivalent derivatives of melarsoprol, such as melarsen oxide and phenylarsine, are highly active even in relatively low concentrations, while its pentavalent derivatives are considerably less active, and its nonarsenical chemical constituents are completely inactive against *T.b.* [69, 88].

Melarsoprol was introduced to replace tryparsamide, another arsenical, and the drug regimens were not supported by pharmacokinetic studies [91]. After recent assessment of pharmacological properties and profile of melarsoprol, the treatment schedule has been improved. Much of the drug has been found to bind to plasma protein with a mean serum half-life of active metabolite of 3.5–3.8 h and a very slow elimination time from the CSF with a half-life of 120 h [92]. The drug regimen used nowadays is a standardized 10-day course with 2.2 mg/kg once a day instead of 3 series of 4 intravenous injections (at a dose of 3.6 mg/kg), with interval of 10 days between each series, as previously adopted [32, 89]. This drug regimen reduces drastically the time of exposure to melarsoprol but fails to show improvements of the severe side effects of this drug, particularly the lethal reactive encephalopathy [93].

In the field, failures of HAT treatment with melarsoprol have reached 30% of the treated cases in several foci [21, 22]. Most parasites selected for resistance to melamine-based arsenicals in the laboratory and several parasites isolated from relapse cases in the field have been shown to have lost the P2 aminopurine transporter [14, 87]. However, trypanosomes from which this transporter has been removed are only marginally less sensitive to melamine-based arsenicals compared to wild-type cells [94]. This suggests the existence of secondary routes of uptake and indicates that the loss of P2 transporter must be coupled with the loss of these secondary routes for high-level resistance [14]. Melarsoprol resistance has also been shown in trypanosomes with ectopic overexpression of the *tbmrpa* gene that encodes a P-glycoprotein type pump [14].

#### 4.1.4. Eflornithine

Eflornithine (D,L-a-difluoromethylornithine) is an analogue of the amino acid ornithine first developed as a potential antineoplastic agent [14]. This drug is efficient against *T.b. gambiense* but not *T.b. rhodesiense* [31].

Eflornithine has similar affinity for both the mammalian and trypanosomal polyamine biosynthetic enzyme ornithine decarboxylase (ODC) and acts as an inhibitor of this enzyme [95]. *T.b. gambiense *ODCs are degraded and replenished much more slowly than in the mammalian counterpart, and therefore, eflornithine deprives trypanosomes of polyamine synthesis for a prolonged period compared with mammalian cells. This polyamine biosynthesis inhibition is accompanied by an increase in cellular levels of S-adenosyl methionine, which causes inappropriate methylation of proteins, nucleic acids, lipids, and other cell components [14, 95, 96]. At variance with *T.b. gambiense*,* T.b. rhodesiense* present a rapid turnover of that enzyme, rendering eflornithine noneffective against this parasite [97]. A diminution of trypanothione levels was also observed after eflornithine treatment [14, 98], and this may increase the susceptibility of *T.b. gambiense *to oxidative stress and other immunological insults.

Eflornithine passive diffusion across the plasma membrane was proposed to account for the uptake of eflornithine in both bloodstream forms of *T.b. *[99] even if evidence from genomic studies suggests the presence in *T.b. *of genes encoding amino acid transporters which could carry eflornithine [100, 101]. Doses beyond 100 mg/kg of eflornithine given *per os* fail to increase the drug level in the plasma, suggesting that the drug is accumulated by a saturable transporter [102]. It is also probable that a transporter carries the drug across the BBB, from where the “y system” (the more important cationic amino acid transport system in mammals) takes over [102, 103].

Little serum protein binding of eflornithine occurs, and, accordingly, the mean half-life in plasma following intravenous injection of eflornithine is about 3-h, with up to 80% of the drug excreted unchanged in urine after 24 h [104]. Thus, the drug regimen is very fastidious, as large doses are given by prolonged intravenous infusion [22, 32].

Eflornithine resistance of *T.b.* procyclic forms has been shown to be related to a reduction of drug uptake [99, 105], suggesting that resistance could be related to loss or changes of eflornithine transport into cells.

### 4.2. New Combination Therapy and Drugs in Clinical Trial

#### 4.2.1. Nifurtimox-Eflornithine Combination Therapy (NECT)

Nifurtimox is a drug used to treat another trypanosomal illness, Chagas disease or American trypanosomiasis caused by *T. cruzi*. The organization “Medecins Sans Frontières” has conducted 2 sequential clinical drug-combination studies at HAT treatment sites in northern Uganda from 2001 to 2004, which have reported that NECT is highly effective and well tolerated [76, 79]. NECT was added to the “WHO Essential Medicines List for the treatment of second-stage Gambian HAT” in April, 2009, on the basis of key advantages over the previous therapeutic options such as high efficacy and good safety profile consistently observed [6].

NECT is easier to administer, requires fewer human and material resources compared with eflornithine monotherapy, and currently stands as the most promising first-line treatment for second-stage Gambian HAT [106]. NECT requires 14 intravenous infusions of eflornithine over 7 days and oral administration of nifurtimox 3 times per day for 10 days, while eflornithine monotherapy requires 56 intravenous infusions over 14 days [76, 106]. However, the training needs for NECT are still considerable in treatment centers that have not yet used eflornithine [106].

NECT has been suggested to be less susceptible to generate parasitic resistance, as this treatment strategy combines two drugs with different modes of action [13].

#### 4.2.2. Diamidines

Several thousands of diamidine derivatives with a broad range of trypanocidal activity and surprisingly diverse pharmacokinetic profiles have been developed [13]. One of these derivatives approved by the US FDA for the treatment of *Pneumocystis jiroveci* pneumonia, pafuramidine (DB289), demonstrated equal efficacy and less overt toxicity with/than pentamidine in a multicenter phase 3 trial involving 273 HAT patients.

Interestingly, some aza analogs of DB289 have shown similar *in vitro* profiles against different *T.b*. strains, melarsoprol- and pentamidine-resistant lines, and a P2 transporter knockout strain (AT1KO) [107]. Some of these compounds, as DB75, show a higher trypanocidal activity [108], and others as DB868 have been reported not only to kill the trypanosomes in the peripheral organs and in the blood compartment, but also, interestingly, to cross the BBB in levels sufficient to kill trypanosomes in the HAT stage 2 mouse model, suggesting efficiency in both stages of the infection [107, 109]. CPD-0802, a compound of this group, is currently under consideration for clinical development for stage 2 HAT [13].

#### 4.2.3. Nitroheterocycles

The discovery in *T.b*. metabolism of an unusual bacterial type 1 nitroreductase enzyme capable of the reductive activation of nitro compounds, that is not found in mammals [74], has “added impetus to the quest as did the introduction of novel nitroheterocycles into clinical trials for tuberculosis, anaerobic protozoan and helminth infections” [13]. Among the numerous compounds tested, fexinidazole showed to be efficient with oral dosing in the mouse model of stage 2 HAT, and the drug proved be metabolized to trypanocidal sulphoxide and sulphone metabolites. In 2009, fexinidazole entered the phase I clinical trials which are presently ongoing [13].

### 4.3. Emerging Challenges for Vaccine Development

The emerging challenges for the development of a vaccine against African trypanosomiasis were recently reviewed [29, 110], and in this section, they will be briefly discussed. The cell surface of the procyclic and epimastigote forms of *T.b.* (found in the fly) is covered with an invariant glycoprotein coat composed of about 10 million copies of two isoforms of a protein named procyclin. These isoforms, named accordingly to their amino acid repeats in their C-termini, are EP-procyclin (which has 22–30 Glu-Pro repeats), and GPEET-procyclin (which has 5-6 Gly-Pro-Glu-Glu-Thr repeats followed by 3 EP repeats), [4, 111]. Both protein isoforms are attached to the membrane via GPI anchors [112, 113]. When epimastigotes differentiate into the metacyclic form (the form inoculated by the fly), the EP-procyclin and GPEET-procyclin coat is replaced by about 10 million copies of a single VSG. Once in the host bloodstream, the parasite keeps the metacyclic VSGs for up to 7 days and then switches to the expression of nonmetacyclic VSGs [112, 114]. In the bloodstream parasites, about 1,000 genes are coding for VSGs [115], thanks to which African trypanosome species escape the immune response of the hosts by readily switching their surface coat, sequentially expressing different forms of VSG at a rate of 10^−2^ to 10^−7^ switches/doubling time of 5−10 h [4, 113, 116]. Such escape mechanism confirms the adaptation of the parasite to its hosts and constitutes the main difficulty for the development of a vaccine against African trypanosomiasis and also because of the incomplete understanding of the control and execution of this immune evasion strategy in trypanosomes [114, 117, 118].

The VSG coat challenge has led to the question of the development of a non-VSG-based vaccine. African trypanosomes express numerous nonvariable surface antigens. In the recent years, many non-VSG candidates have been used for experimental vaccination schemes for trypanosomiasis; most reports prove promising, but not a single strategy was effective enough for the development of an effective vaccine [110]. Of particular interest has been the flagellar pocket, an organelle specialized in endocytosis and exocytosis containing relatively well-conserved receptors [119], and cytoskeleton proteins, as an interesting group of nonvariable antigens [120]. The main pitfall that might explain the failure of all these strategies was suggested to be the fact that immunization against these proteins might never result in significant B cell memory-based protection in experimental model systems that are characterized by an excessively high parasite burden early on in infection, as most of the models used up to know for vaccine development [110]. In order for a vaccine targeting trypanosomes to act, it should have the ability to eliminate all circulating trypanosomes before they trigger mechanisms of B cell memory suppression or destruction [45]. Thus, these parasites seem, up to now, to have always been able to modulate the B cell memory response in their advantage, impeding the B cell response that aims to eliminate them, rendering further more difficult the realization of a vaccine.

Interestingly, a positive note derives from research attempting another approach: the development of vaccines aimed to reduce *T.b.* transmission through immunization against insect parasite stages which express an invariant glycoprotein coat, that is, blocking the parasitemia onset in the host, as the successful antitick vaccine [110]. Several antigens have been already proposed as candidates for such experimental vaccination schemes and are being tested [110].

## 5. The Potential of Medicinal Plants in Trypanosomiasis Management

### 5.1. Medicinal Plants as Alternative Drugs

Interest in higher plant extracts exhibiting antimicrobial activity has increased in recent years, and several reports on this subject have been published. Indeed, the use of and search for drugs derived from plants have accelerated in recent years [121–128], whereby ethnopharmacologists, botanists, microbiologists, and natural-product chemists are combing the earth for phytochemicals and “leads” which could be developed for the treatment of various ailments. WHO has estimated that 80% of the population of developing countries relies on traditional medicines, mostly plant drugs, for their primary health care needs [129–132]. For instance, the use of herbs and medicinal plant products has become a mainstream phenomenon over the past two decades in many countries, where herbs and phytomedicines (herbal remedies) have become one of the fastest growing segments in retail pharmacies and supermarkets [121, 128]. It is of no denying that medicinal herbs now constitute the most rapidly growing segment of the total US pharmaceutical market and are now used by approximately 20% of the population [133, 134]. Available reports tend to show that about 25% of all prescriptions sold in the US are from natural products, while another 25% are from structural modifications of a natural product [134, 135]. In other reports [136, 137], it is proposed that 3 in 10 Americans use botanical remedies in a given year giving rise to a whole new industry referred to as “nutraceuticals” and currently, 20,000 herbal products are available in this country [134, 138].

Indeed, it is clear from available literature that modern pharmacopoeia still contains at least 25% drug derived from plants, and many others, which are synthetic analogues, built on prototype compounds isolated from plants [125, 135]. Despite the availability of different approaches for the discovery of therapeuticals, natural plant products still remain as one of the best reservoirs of new structural types. Concurrently, many people in developing countries have begun to turn to alternative therapies as cheap sources of complex bioactive compounds and evidence of the beneficial therapeutic effects of these medicinal herbs is seen in their continued use [124, 127, 134, 139]. The importance of medicine of natural product molecules lies not only in their pharmacological or chemotherapeutic effects, but also in their role as template molecules for the production of new drug molecules. It is of no denying that knowledge gained from the use of medicinal herbs and their active ingredients has served as the foundation for much of modern pharmacology, and many modern drugs have their origin in ethnopharmacology. Additionally, the development of modern chemistry has permitted the isolation of chemicals from medicinal herbs which have served as drugs or starting materials for the synthesis of many important commercially important drugs used today [139, 140]. Drugs such as aspirin, digitalis, morphine, metformin, and quinine amongst others were all originally isolated or synthesized from materials derived from plants [122, 141].

Medicinal plants, unlike pharmacological drugs, commonly have several chemicals working together catalytically and synergistically to produce a combined effect that surpasses the total activity of the individual constituents. The combined action of these substances increases the activity of the main, medicinal constituent by speeding up or slowing down its assimilation in the body. Secondary substances from plant origins might increase the stability of the active compound(s) or phytochemicals, minimize the rate of undesired side effects, and have an additive, potentiating, or antagonistic effect [125, 126].

With the exception of antimalarials and as mentioned above, there are currently only four drugs approved to treat HAT. However, eflornithine and pentamidine are ineffective against sleeping sickness caused by *T.b. rhodesiense*. Treatment with melarsoprol, the only generally effective first-line drug, required lengthy parenteral administration and can result in up to 10% mortality. Additionally, the toxicity and the upsurge in the number of patients failing to respond to melarsoprol because of drug resistance reflects the need for discovery of new chemotherapeutic agents against HAT [142]. To this effect, the insufficiency of current therapies for the treatment and management of trypanosomiasis, combined with both a lack of trust in conventional medical treatment and an inability of the economy to absorb the cost of pharmaceuticals, have created a growing public interest in alternative natural drugs from botanicals.

### 5.2. Phytotherapy for HAT

Drug-screening activities from plants have started decades back, and an emerging number of studies have now been developed and reported so far to discover drugs from medicinal plants that can help to combat trypanosomiasis. The main aim has been geared towards alternatives to conventional drugs with fewer side effects but greater effectiveness.

A plethora of studies has been conducted to investigate the effect of some traditionally used medicinal plants in alleviating the cellular changes *in vivo *produced during the *T.b. brucei *infections of rats. Traditional knowledge was the basis for the selection of plants, and one study included *Momordica balsamina *pulp, *Aloe vera *pulp, *Annona senegalensis*,* Securidaca longipenduculata *root, and root bark. On the basis of folk medicines, they were claimed to possess antiprotozoal activity and alleviate one or many of the clinical symptoms such as intermittent fever, immunosuppression, anemia, jaundice, and hepatomegaly commonly associated with trypanosomiasis. Interestingly, it was found that these plants had the potential in the management of HAT due to the fact that *T.b. brucei*. *Momordica balsamina*, and *S. longipenduculata *were found to possess the highest potential, since they are able to control anemia by resisting sudden drop in packed cell volume values [143].

In another study, it was showed that the extracts of *Hymenocardia acida* stem bark exhibited significant trypanocidal activity, whereas *Gardenia erubescens *and *Lophira lanceolata *were effective at minimum inhibitory concentration (MIC) of 20 mg/mL [144]. Nigerian plants were also evaluated *in vitro *for trypanocidal activity against *T.b. brucei *and *T. congolense *at concentrations of 4 mg/mL, 0.4 mg/mL, and 0.04 mg/mL. It was found that extracts of *Khaya senegalensis*,* Piliostigma reticulatum*,* Securidaca longepedunculata*, and *Terminalia avicennoides *were strongly trypanocidal to both organisms while extracts of *Anchomanes difformis*,* Cassytha spp*,* Lannea kerstingii*,* Parkia clappertioniana*,* Striga spp*,* Adansonia digitata*, and *Prosopis africana *were trypanocidal to either *T.b. brucei *or *T. congolense*. *Kigelia africana*, from Kenya, was also evaluated *in vivo*, and it was found that the dichloromethane fruits extract of *K. africana *tested at a dose of 2000 mg/kg was effective, curing 60% of the Swiss white mice that had previously been inoculated with *T.b. rhodesiense *KETRI 3798 [145, 146].

In another investigation, *Scoparia dulcis *was evaluated on the population of immune cells during a 28-day experimental *T. brucei *infection in rabbits. The result obtained showed that infection resulted in an initial rise in both total WBC and the absolute number of circulating lymphocytes followed by a progressive decrease in total WBC and all WBC subtypes (lymphocytes, monocytes, and granulocytes) although the percentage of lymphocytes remained consistently higher than normal throughout the study period. These changes were consistent with the development of trypanosome-induced immunosuppression in their mammalian host, and interestingly, treatment with *S. dulcis *at a daily oral dose of 25 mg/Kg body weight was found to significantly reduce the severity of the observed lesions when compared with untreated infected animals. Thus, *S. dulcis *was classified as a potential herb that had demonstrated significant potency in protecting against the parasite-induced decrease in the population of immunologically active cells [147].

From the above key investigations, it is clear that these findings provide strong evidence of the potential beneficial effects of phytotherapy in the traditional management of trypanosomiasis which could be subsequently developed into a cost-effective alternative drug to complement treatment of trypanosomiasis.

### 5.3. Possible Mechanism of Phytochemicals against Trypanosomiasis

In many investigations conducted so far, it was found that the plant parts differ significantly in their activity. The differences observed in the antimicrobial evaluation suggest the susceptibility of the test microorganism to various secondary metabolites present in these medicinal plants. In general, discussions pertaining to anti-Trypanosoma agents from plants center on plant secondary metabolites, that is, nonubiquitous constituents with no known essential role in the plant's metabolism. However, it has been postulated that bioactive plant secondary metabolites may play a role in chemical defense mechanisms and are likely molecules for the antiparasitic agents in these plants [125, 126].

Recently, it has been postulated that the enormous diversity of chemical structures found in these plants are not waste products but specialized secondary metabolites involved in the relationship of the organism with the environment, for example, as attractants of pollinators, signal products, defensive substances against predators and parasites, or in resistance against pests and diseases [140]. Indeed, the composition of these secondary metabolites in turn varies from species to species, climatic conditions, and the physiological state of developments of the endemic plants [148]. Available reports tend to show that alkaloids and flavonoids are the responsible compounds for the antimicrobial activities, and anti-Trypanosoma in higher plants [149]. Moreover, it is also claimed that secondary metabolites such as tannins and other compounds of phenolic nature are classified as active antimicrobial compounds [150, 151].

Nonetheless, several investigations tend to suggest that it is often difficult to speculate and decipher the exact mode of action by which these plants extracts exhibit their trypanocidal action. Indeed, the possible mechanisms by which these plants extracts and phytochemicals therein carry out this role remain a subject of great speculations and debate in the scientific community. Several possible mechanisms working separately or in concert may account for the observed effect [147].

In one study, it was suggested that different phytocompounds could be responsible and operate in a synergistic effect for the observed antitrypanocidal activities. Interestingly, preliminary phytochemical screening of potent plants against trypanosome showed the presence of biological known active compounds such as saponins, tannins, flavonoids, and alkaloids in the crude plant extracts tested. Several authors have also identified or isolated tannins and phenolic compounds, flavonoids, and alkaloids in plants that showed significant trypanocidal activities [152].

Accumulated evidence also suggest that many natural products exhibit their trypanocidal activity by virtue of their interference with the redox balance of the parasites acting either on the respiratory chain or on the cellular defenses against oxidative stress. For instance, the observed trypanocidal activity of *K. africana *extract was justified due to the increase of oxygen consumption and stimulation of hydrogen peroxide production in the protozoan cell. Trypanosoma do not have the same biochemical mechanism as mammalian cells for dealing with excess peroxide and consequent oxygen free radicals [146]. Furthermore, it is proposed that natural products possess structures capable of generating radicals that may cause peroxidative damage to trypanothione reductase that is very sensitive to alterations in redox balance. It is also known that some agents act by binding with the kinetoplast DNA of the parasite.

On the other hand, the result of [153] has clearly indicated that different solvent extracts of the same plant may exhibit different trypanocidal activity just as extracts of different parts of the same plants. Therefore, the statement that a plant is trypanocidal or not should be taken within the context of the solvent used and the parts investigated. On the other hand, out of the 40 plant extracts tested by [154, 155], the dichloromethane extract from stem bark of *Warburgia salutaris *(claimed to be used against many pathologies in many parts of Africa) was found to exhibit the most potent trypanocidal activity. The trypanocidal activity was suggested to be due to the drimane sesquiterpenoids (warburganal and polygodial). Concerning the mechanism, it was proposed that the two sesquiterpene aldehydes, warburganal and polygodial, formed covalent bonds with amino groups of proteins and affect a vast number of cellular activities. In another groundbreaking *in vitro *study, the authors were able to isolate the pregnane glycosides from genus Caralluma (*C. Penicillata*,* C. tuberculata*, and *C. russelliana*) and evaluated for the trypanocidal activity. It was found that the penicilloside E to possess the highest anti-Trypanosoma activity followed by caratuberside C, which exhibited the highest selectivity index [142].

Studies have shown that it is probable that the etiology of trypanosome-induced leucopenia in rabbit may be similar to the case with trypanosome-induced anemia. There has been striking indications that the onset of anemia in HAT may be strongly related to disruption of erythrocyte membrane caused directly by parasite attack on red cells. It has also been suggested that products secreted by the parasite may play a significant role in the disruption of red cell membrane. Reduction in red cell membrane sialoglycoprotein secondary to elevated activity of plasma sialidases promotes the rapid destruction of erythrocytes. A role for parasite and macrophage-derived free radicals and proteases in the pathogenesis of trypanosome-induced anemia has also been postulated [147]. The possibility that *S. dulcis *or certain components of the herb may help stabilize the membrane of blood cells cannot be outrightly dismissed. Specifically, it is not out of place to suggest that the antioxidant or free radical scavenging properties of *S. dulcis* may play vital roles in this regard especially against the backdrop of the role of free radicals in the pathogenesis of *T. brucei *infection and also probable that increased production of blood cells helps in replenishing of these cells. In the absence of any evidence of possible trypanocidal activity for the herb, it does not seem an attractive option to speculate that the higher level of immunological cells in treated animals could be due to the destruction of the parasite by agents native to the plant [147]. On the other hand, [156] have showed that *Psidium guajava* leaf extract has trypanocidal properties and has attributed these effects in parts to the broad antimicrobial and iron chelating activity of flavonoids and tannins, respectively. They have also proposed that iron chelation is an effective way of killing trypanosomes and the prime target is the enzyme ribonucleotide reductase whose activity is central to DNA synthesis prior to cell division as depicted in trypanosomiasis infection.

Moreover, a plant with high *in vitro *trypanocidal activity may have no *in vivo *activity and vice versa because of peculiarities in the metabolic disposition of the plant's chemical constituents. Therefore, plants found to be active in the above-mentioned investigations must be tested *in vivo *and tested clinically before a definite statement can be made on their trypanocidal potentials [145].

## 6. General Conclusions

Once neglected, HAT has returned in the center of the attentions of the scientific community, resulting in the recent reversal of the trend of cases which was increasing. However, as illustrated in Figure 1, studies have shown that in the last 30 years, HAT cases occurred more often in countries with conflict, high political terror, or civil war, with an interval as long as 10 years between the start of conflict events and a peak in incidence [12], and considering that unfortunately, these events still are current in HAT endemic regions, the risk of future epidemics is considerable. Fortunately, a new approach for vaccine development targeting the insect parasite stages is being tested and bear higher chances of success than the precedent approaches. In addition, powerful molecular tools analyzing inflammatory mediators are also proving very efficient for HAT diagnostic staging and followup. Therefore, even if in the field, the problem of proper diagnostic and staging, together with the posttreatment followup remain the adaptation of such molecular techniques to field conditions that will solve these gigantic difficulties.

The research re-engagement also has produced promising antitrypanocidal molecules from the diamidine and nitroheterocycle pharmacological classes which are under further tests and considered for clinical trials. Prodrugs able to cure both phases may even have been found. Overall, developing new drugs to replace the very toxic ones still in use is crucial, together with more reliable tools for disease staging and treatment followup. In the field, even if only one drug, eflornithine, has been developed in the last 50 years, a new combination therapy involving that drug and another one developed for Chagas disease, nifurtimox, is proving a good replacement for the highly toxic melarsoprol which was previously the exclusive drug able to cure stage 2 patients. But this combination of antitrypanocidal drugs appears to be efficient only against *T. gambiense*, leaving the treatment of *T. rhodesiense* stage 2 patients to the latter arsenic-derived drug. To this effect, the active principles of traditional pharmacopoeia and medicinal plants of African countries have been scrutinized over the past decade as prospective alternative trypanocides due to less toxicity and side effects. Indeed, the recent quest for novel anti-HAT pharmacophore has been geared mainly towards traditional medicines, local knowledge, and ethnopharmacology. Phytotherapies, besides their traditional and holistic values, also hold great public and medical interest worldwide as cheap sources of nutraceuticals with new template compounds of high activity and selectivity.
